# A side-by-side comparison of the solidification dynamics of quasicrystalline and approximant phases in the Al–Co–Ni system

**DOI:** 10.1107/S2053273318017114

**Published:** 2019-02-06

**Authors:** Insung Han, Xianghui Xiao, Haiping Sun, Ashwin J. Shahani

**Affiliations:** a Department of Materials Science and Engineering, University of Michigan, Ann Arbor, Michigan 48109, USA; b X-ray Science Division, Advanced Photon Source, Argonne National Laboratory, Lemont, Illinois 60439, USA; c Michigan Center for Materials Characterization, University of Michigan, Ann Arbor, Michigan 48109, USA

**Keywords:** quasicrystals, approximant phases, synchrotron radiation, nucleation and growth

## Abstract

Quasicrystals and their approximants have triggered widespread interest due to the challenge of solving their complex crystal structures as well as their possibly exceptional properties. The structural motifs of approximants are similar to those of the corresponding quasicrystals, but to what extent are their crystallization pathways the same? Unfortunately, there have been very few *in situ* experimental investigations to answer this question. Here, by leveraging the high penetrating power of hard X-rays, synchrotron-based X-ray tomography was conducted in order to capture the nucleation and growth of a decagonal quasicrystal and its related approximant. The combination of data-driven computational analysis with new thermodynamic databases allowed the characterization, with high precision, of the constitutional and kinetic driving forces for crystallization. The experimental results prove that the growth of both crystals from a liquid is dominated by first-order kinetics. Nevertheless, and somewhat surprisingly, significant differences were observed in their rates of nucleation and growth. The reasons for such divergent behaviours are discussed in light of contemporary theories of intermetallic crystallization.

## Abstract 2

The nucleation and growth dynamics of a decagonal quasicrystal and its approximant ‘*X*’ phase are considered. Various similarities and differences are elucidated.

## 1. Introduction   

Soon after the discovery of icosahedral quasicrystals (Shechtman *et al.*, 1984 ▸; Shechtman & Blech, 1985 ▸), it was discovered that quasiperiodicity is not limited to icosahedral phases. For instance, decagonal quasicrystals (d-QCs) possess quasiperiodic, 2D atomic layers that are stacked periodically in the perpendicular direction (Bendersky, 1985 ▸). At the mesoscale, d-QCs show a columnar prismatic morphology along the direction of periodic stacking (Stadnik, 2012 ▸). A consequence of this unique crystallographic anisotropy is strongly anisotropic transport properties, *e.g.* electrical resistivity and thermal stability (Shibuya *et al.*, 1990 ▸; Edagawa *et al.*, 1996 ▸; Stadnik, 2012 ▸). Among the discovered d-QCs, Al–Co–Ni d-QCs have been the most thoroughly investigated so far (Tsai *et al.*, 1989 ▸; Yamamoto *et al.*, 1990 ▸; Ritsch, 1996 ▸; Ritsch *et al.*, 1998 ▸; Yuhara *et al.*, 2004 ▸). Within the Al–Co–Ni d-QC phase, a wide variety of polymorphism on the quasiperiodic planes has been reported (Hiraga *et al.*, 2001*a* ▸,*b* ▸; Cervellino *et al.*, 2002 ▸). Closely related to the d-QC phases are a number of so-called approximant phases that have been described by many researchers (Honal *et al.*, 1998 ▸; Hiraga *et al.*, 2001*b* ▸; Steurer, 2001 ▸; Katrych *et al.*, 2006 ▸; Mihalkovič & Widom, 2007 ▸; Smontara *et al.*, 2008 ▸; Dolinšek *et al.*, 2009 ▸; Katrych & Steurer, 2009 ▸; Fleischer *et al.*, 2010 ▸; Gille *et al.*, 2011 ▸). To be considered as an approximant phase of the d-QC, two conditions need to be met: (i) the substructures (or structural tiles) of the approximant must be similar to the d-QC. Hence, the approximant phase should have atomic decorations which resemble those of the aperiodic plane of d-QCs. (ii) The approximant phase must consist of periodically stacked atomic layers with a periodicity length almost identical to that of the d-QC (Dolinšek *et al.*, 2009 ▸; Gille *et al.*, 2011 ▸). Periodic stacking of two (Zurkirch *et al.*, 1998 ▸; Cervellino *et al.*, 2002 ▸; Henley *et al.*, 2002 ▸; Mihalkovič *et al.*, 2002 ▸; Katrych *et al.*, 2006 ▸; Smontara *et al.*, 2008 ▸) or four (Gu *et al.*, 2006 ▸, 2007 ▸; Mihalkovič & Widom, 2007 ▸; Fleischer *et al.*, 2010 ▸) atomic layers is commonly observed in the d-QCs and their approximant phases along the 〈00001〉 axis and 〈010〉 axis, respectively. Therefore, the *approximant phase of d-QCs can be more broadly defined as a crystal structure sharing similar motifs of d-QCs*, and can include a number of compositional variants.

### 1.1. Complex intermetallics in the Al–Co–Ni system   

Below, we survey a few noteworthy approximant phases of Al–Co–Ni d-QCs. One of the well-known d-QC approximant phases is the binary Al_13_Co_4_ phase. Orthorhombic (*o*-) and monoclinic (*m*-) Al_13_Co_4_ are stable above 750 K. Both variants share the same elementary cluster, a pentagonal bipyramid that is composed of alternate flat and puckered atomic layers. The stacking of these alternate layers gives rise to a four-layer periodicity along the *b* axis of the crystal (Mihalkovič & Widom, 2007 ▸). It is known that the *m*- and *o*-Al_13_Co_4_ phases are entropically stabilized by disorder in the puckering of Co atoms from the atomic layer, as well as vacancy disorder, tiling disorder and thermal vibration (Mihalkovič & Widom, 2007 ▸). Investigators also report another related approximant phase, *o*′-Al_13_Co_4_, which has a similar atomic arrangement, stacking sequence, vacancy disorder and high-temperature stability as the *m*- and *o*-Al_13_Co_4_ phases (Fleischer *et al.*, 2010 ▸).

**Figure 1:**
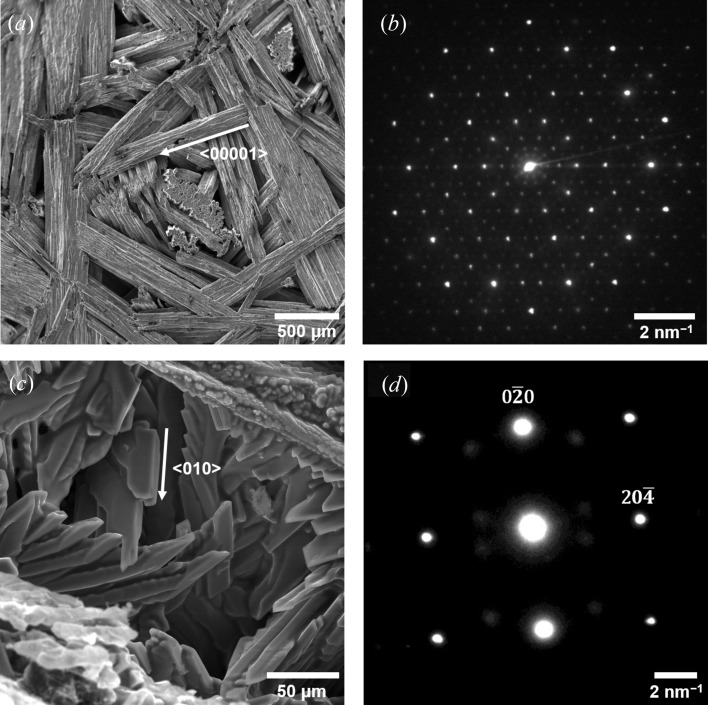
SEM images and electron diffraction patterns of (*a*), (*b*) Al–Co–Ni d-QC and (*c*), (*d*) the *X* phase. The sample preparations for the approximant *X* phase are described in Section 2, while those for the d-QC are given in our previous work (Han *et al.*, 2017 ▸). The arrow in (*a*) indicates the periodic 〈00001〉 direction in the d-QC and the arrow in (*c*) represents the 〈010〉 direction (or *b* axis) of the *X* phase. The diffraction pattern, reprinted from Han *et al.* (2017 ▸), in (*b*) shows tenfold symmetry along 〈00001〉, while that in (*d*) represents a monclinic crystal structure (Pearson symbol *ms*26) along the [201] zone axis.

Beyond the binary approximant phases, investigators have found ternary approximants of Al–Co–Ni d-QCs. For instance, the ternary *W* phase is stable at high temperature in the range of 1173 to 1343 K (Katrych & Steurer, 2009 ▸) and has a monoclinic unit cell (Hiraga *et al.*, 2001*a* ▸,*b* ▸). The approximate compositions of the *W* phase are in the range of 0 to 11 at.% of Ni and 68 to 74 at.% of Al (Katrych & Steurer, 2009 ▸). Through high-resolution transmission electron microscopy (HRTEM) and high-angle annular dark-field scanning transmission electron microscopy (HAADF-STEM), investigators have demonstrated that the decagonal cluster that forms the *W* phase can be found in the various structural modulations of Al–Co–Ni d-QCs (Hiraga *et al.*, 2001*b* ▸). The *W* phase has a unit cell composed of four layers along the *b* axis and thus pseudo-pentagonal symmetry can be obtained in the electron diffraction pattern with the incident beam parallel to the *b* axis (Hiraga *et al.*, 2001*b* ▸; Katrych & Steurer, 2009 ▸). The layer of 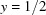 along the *b* axis is a mirror plane, and the layers of 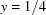 and 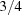 have a puckered structure similar to the aforementioned Al_13_Co_4_ polymorphs. In addition, the ternary *X* phase is a high-temperature approximant phase that can coexist with the four-layer Ni-rich decagonal phase within a broad temperature and composition range (Katrych *et al.*, 2006 ▸). The composition of the ternary *X* phase is given by Al_9_(Co,Ni)_4_. The Co/Ni disorder on transition metal sites contributes to the free energy of the *X* phase in terms of a high configurational entropy, giving rise to its stability at elevated temperatures (Katrych *et al.*, 2006 ▸). The *X* phase consists of two flat atomic layers along the *b* axis; each layer includes distorted pentagonal motifs (Katrych *et al.*, 2006 ▸). Like the *W* phase, the *X* phase has a monoclinic unit cell. It shares a similar, columnar morphology with the d-QC (Fig. 1 ▸) that will be elaborated upon below.

Single-grained QCs and their related approximants can be grown via conventional approaches including Czochralski, Bridgman, floating zone and self-flux methods, which we briefly review here. The Czochralski method uses a small piece of seed crystal and growth occurs atop the seed through incongruent solidification from the molten alloy (*i.e.* solid and melt have different compositions). Through careful control of the temperature gradient, rotation speed and crystal pulling speed (0.1–10 mm h^−1^), large d-Al–Co–Ni crystals of 1 cm in diameter have been obtained (Gille *et al.*, 2005 ▸). In the Bridgman method, nucleation occurs spontaneously rather than on a pre-set seed. The sample is kept at high temperature (above the liquidus) for homogenization; then, the sample is slowly pulled down to pass the temperature gradient zone where crystallization proceeds. With this method, high-quality d-Al–Co–Ni crystals have been grown (Feuerbacher *et al.*, 2003 ▸). The floating zone method uses a polycrystalline feed rod with the same composition as the target crystal. A floating zone (*i.e.* molten zone) is formed in the feed rod by selective heating. As the zone moves upward, crystallization occurs from the bottom to the top of the feed rod (see, *e.g.*, Sato *et al.*, 1998 ▸). Last and simplest of all is the self-flux method, in which crystals are grown by cooling an off-stoichiometric melt below the liquidus temperature. Unlike the other three methods, self-flux does not involve a temperature gradient nor selective heating. Our approach is most similar to the self-flux method yet does not include the final decanting step (Tsai & Cui, 2015 ▸). Further details on our approach are given in Section 2.1.

### 1.2. Crystallization pathways of complex intermetallics   

While their structures have been extensively studied, the growth kinetics of the complex intermetallic phases from a liquid phase have been much less explored. To this end, Steurer has identified in his topical reviews (Steurer, 2017 ▸, 2018 ▸) the problem of quasicrystal growth as one of the still open questions facing the crystallographic community, as of 2018. He asks, ‘How does the 1000th atom find its site in a giant unit cell with thousands of atoms?’ One physically plausible explanation is that the growth (of both QCs and their approximants) occurs not by the attachments of single atoms but rather energetically favourable clusters of atoms that pre-exist in the liquid phase. If this cluster-based argument is to be believed, then one might suppose that the rate of crystal growth slows with increasing cluster size. This is because large clusters require more time to rotate towards the correct orientation (Land & Yoreo, 2000 ▸; Chernov, 2012 ▸; Markov, 2016 ▸); in this way, clusters should overcome a configurational entropy-type barrier in order to incorporate into the solid phase.

Experimental support comes from recent *in situ* studies conducted by Han *et al.* (2017 ▸), who tracked the growth dynamics of a single Al–Co–Ni d-QC in the aperiodic directions. According to Han *et al.* (2017 ▸), the crystallization of the d-QC occurs via first-order kinetics (*i.e.* the solid–liquid interfacial normal velocity *V* scales linearly with the undercooling 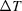). The constant of proportionality between the two is known as the kinetic coefficient (hereafter written as 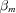, unit cm s^−1^ K^−1^). The kinetic coefficient 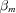 for the d-QC was found to be approximately six to nine orders of magnitude smaller than those of periodic, elemental metallic crystals and two to eight orders of magnitude smaller than those of periodic, intermetallic crystals (excluding approximants) (Han *et al.*, 2017 ▸). Thus, *the kinetic coefficient decreases as the complexity of the growth unit or cluster increases*. An alternate viewpoint that does not rely on the cluster description is offered by Herlach (1994 ▸). He suggests that short-range diffusion is necessary for the atoms to sort themselves out to find the proper sublattice position in a given intermetallic solid. In contrast, the attachment kinetics at the solid–liquid interface of a simple crystal (such as a pure metal) are only collision limited (Coriell & Turnbull, 1982 ▸). Consequently, for diffusion-limited atomic attachment driven growth of intermetallics, the kinetic coefficient should be orders of magnitude smaller as compared with collision-limited growth of simple crystals, since *the relaxation frequency for atomic diffusion* (former case) *is much less than the Debye frequency* (latter case).

Based on the steric argument given by Land & Yoreo (2000 ▸) and Chernov (2012 ▸), one might possibly assume that the kinetic coefficients of QCs and their approximants are the same, owing to their similar structural motifs [condition (i) above]. To test this hypothesis, we captured in real time and at elevated temperature the solidification dynamics of the *X* phase during ‘fast’ X-ray imaging and compared the results against the d-QC reported earlier by our team (Han *et al.*, 2017 ▸). Our subsequent analysis of the time-dependent driving force is made possible due to recent thermodynamic assessments of the Al–Co–Ni system (Wang *et al.*, 2016 ▸; Wang & Cacciamani, 2018 ▸). Somewhat surprisingly, we find significant differences in the nucleation and growth rates between the QC and approximant, the reasons for which are discussed in Section 3.2. Broadly, the d-QC possesses a structural flexibility that is not seen in the approximant phase, owing in large part to the QC’s higher (phasonic) degrees of freedom. To the best of our knowledge, our investigation presents the first ever side-by-side comparison of the crystallization behaviours of a QC and its related approximant, through real-time experiments and corres­ponding thermodynamic calculations.

## 2. Materials and methods   

Despite the decades of research devoted to the study of QCs and their approximant phases, it remains to be determined, through experiment, how exactly they emerge from a liquid phase. This is due, in part, to a lack of real-time data with which to test the kinetic theories introduced above. Worth mentioning is one recent experimental study by Nagao *et al.* (2015 ▸) of grain growth in a d-QC specimen using *in situ* HRTEM. The team observed that grain boundaries migrate through an ‘error-and-repair’-type process, wherein phason strain is introduced and subsequently relaxed to generate ideal quasicrystalline order (Nagao *et al.*, 2015 ▸). However, there is no mention of the interfacial dynamics in the periodic 〈00001〉 direction. In general, while one can extract some information from a time-lapse of 2D images, most growth models make predictions based on a 3D structure. To circumvent these challenges, we have investigated crystallization through 4D (*i.e.* 3D space and time-resolved) synchrotron-based X-ray microtomography (XRT). Our unique experiment provides a new vision on the interfacial dynamics of both QCs and their related approximants, leveraging the benefits of high temporal and spatial resolutions, as will be described in detail below.

### 2.1. Experimental methodology   

The experimental methods follow that of Han *et al.* (2017 ▸). Master alloy samples of composition Al–8at.% Co–8at.% Ni were prepared by vacuum arc remelting (VAR) using high-purity elemental Al (99.999%), Co (99.9%) and Ni (99.9%) at the Materials Preparation Center (MPC) at Ames National Laboratory in Ames, Iowa, USA. The cast Al–8at.% Co–8at.% Ni alloy buttons were cut into cylinders of 1 mm diameter by 5 mm height for the XRT experiment. The cylindrical samples were placed into a boron nitride (BN) sample holder, which is chemically inert at high temperatures and nearly transparent to the incident X-ray beam. The synchrotron XRT experiment was conducted at beamline 2-BM of the Advanced Photon Source (APS) at Argonne National Laboratory in Lemont, Illinois, USA. The sample was first held in a resistive furnace for 5 min at 1293.2 K, which is above the liquidus temperature of Al–8at.% Co–8at.% Ni; this was done to homogenize the melt. Then, X-ray projection images were collected continuously as the sample was rotated at a constant slew velocity of 9° s^−1^ and cooled from 1273.2 K to 1213.2 K at a rate of 1 K min^−1^. The total data acquisition time was thus 1 h. Throughout the 1 h scan, the electron beam was ‘topped up’ every 5 min so that the beam current fluctuation was less than 0.5%. Also, the beam heating effect on the sample should be even less than 0.5%. The molten alloy was contained by a thin Al_2_O_3_ layer, naturally grown by thermal oxidation (*i.e.* no vacuum or inert gas atmosphere was utilized). Prior to conducting the XRT experiment, the furnace temperature was calibrated against other Al-based alloys (*e.g.* Al–Si and Al–Ge) with known liquidus and eutectic temperatures, with beam on. The X-ray projection images were collected at a rate of 50 Hz using a polychromatic ‘pink’ beam centred at 27 keV. A PCO Edge 5.5 CMOS camera optically coupled with a 20 µm-thick LuAg:Ce scintillator was used for data collection. The field of view (FOV) measured 2560 by 800 pixels along the specimen *x* and *z* directions, respectively, with a pixel size of 0.65^2^ µm^2^. One thousand projections were recorded for each 180° rotation of the sample with an exposure time of 14 ms, resulting in a temporal resolution of 20 s between successive 3D reconstructions. The synchrotron XRT projection data are stored on the Materials Data Facility (Blaiszik *et al.*, 2016 ▸) and available online at http://dx.doi.org/10.18126/M2K910.

**Figure 2:**
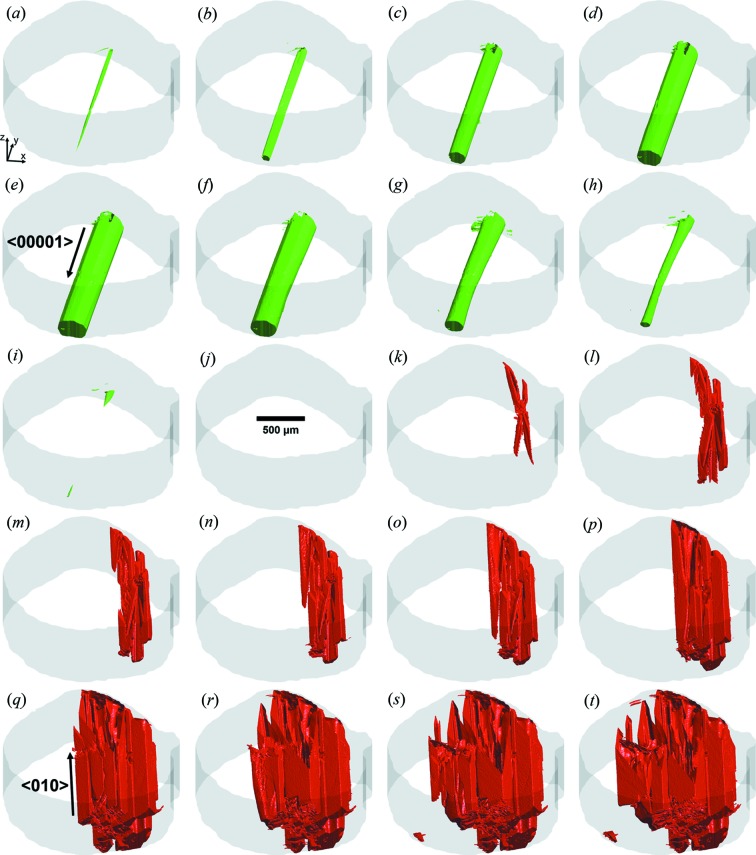
3D reconstructions of (*a*)–(*e*) Al–Co–Ni d-QC growth, and (*f*)–(*j*) its dissolution (in green), followed by (*k*)–(*t*) *X*-phase crystallization (in red) during continuous cooling (1 K min^−1^). The *z* axis in the specimen frame points along the rotation axis of our cylinder sample. Temperatures and times are as follows: (*a*) 1259.8 K (800 s), (*b*) 1259.2 K (840 s), (*c*) 1257.2 K (960 s), (*d*) 1253.2 K (1200 s), (*e*) 1247.8 K (1520 s), (*f*) 1243.5 K (1780 s), (*g*) 1238.5 K (2080 s), (*h*) 1235.2 K (2280 s), (*i*) 1233.8 K (2360 s), (*j*) 1233.8 to 1227.2 K (2360 to 2760 s), (*k*) 1227.2 K (2760 s), (*l*) 1226.8 K (2780 s), (*m*) 1226.5 K (2800 s), (*n*) 1226.2 K (2820 s), (*o*) 1225.8 K (2840 s), (*p*) 1224.5 K (2920 s), (*q*) 1220.8 K (3140 s), (*r*) 1218. 8 K (3260 s), (*s*) 1217. 8 K (3320 s) and (*t*) 1215.5 K (3460 s). The times given in parentheses are with respect to the start of the XRT experiment at 1273.2 K (0 s). A thin grey layer indicates the Al_2_O_3_ protective skin of the molten alloy sample that was grown naturally by thermal oxidation. We observe the nucleation and growth of a *single* d-QC at high temperatures and *multiple* *X*-phase crystals at lower temperatures (see Section 3.1).

Our *in situ* cooling experiment reveals three distinct phase transformations: (i) growth of the d-QC phase from 1259.8 K to approximately 1247.8 K, (ii) complete dissolution of the same phase from 1247.8 K to 1233.8 K, and lastly (iii) growth of an approximant *X* phase from 1227.2 K to the end of the experiment at 1213.2 K. See also Fig. 2 ▸ for 3D snapshots of the microstructural evolution. Processes (i) and (ii) are discussed in our earlier article (Han *et al.*, 2017 ▸). In brief, (i) growth of the d-QC occurs until the liquid phase is depleted enough in Co and Ni to reach the liquidus composition (Yokoyama *et al.*, 1997 ▸). The Al enrichment and Co, Ni depletion during the d-QC solidification were also noted by Guo *et al.* (1999 ▸) at the growth front of a single d-QC. Subsequently, the remaining liquid becomes Al rich – which is likely due to gravity-induced segregation and Al rejection from regions outside the FOV – so that the d-QC is no longer in equilibrium with the Al-rich liquid and hence (ii) it dissolves. Our interest in the present study is to elucidate (iii) the growth process of the approximant phase and compare it with that of the d-QC phase observed at higher temperatures (i). To our advantage, the observed solidification of both solid phases (d-QC and *X* phase) is non-congruent, giving rise to strong absorption contrast between solid and liquid in the XRT experiment.

Further microscopy was carried out at the Michigan Center for Materials Characterization [(MC)^2^], Ann Arbor, Michigan, USA, in order to determine the identities of the above two solid phases seen via XRT (and particularly that of the approximant). Details of electron microscopy done on the d-QC phase are given in Han *et al.* (2017 ▸). Here, we focus on characterizations of the approximant phase. Although we were not able to re-use the same sample investigated via XRT, we replicated the growth conditions on another sample of nominal composition Al–8at.% Co–8at.% Ni. This sample was cooled in a bench-top muffle furnace from 1273.2 K to 1173.2 K at a rate of 1 K min^−1^, and quenched to obtain the fully grown approximant *X*-phase crystals. Then, electron micrographs were taken with a Tescan MIRA3 FEG scanning electron microscope with an accelerating voltage of 30 kV. A representative image is shown in Fig. 1 ▸(*c*). For transmission electron microscopy (TEM), regions rich in the approximant phase in the alloy sample were selected and ground into powder; the powder was diluted in ethanol and transferred onto a standard 3 mm-diameter Cu grid with carbon support thin film for TEM observation. TEM images and electron diffraction patterns were recorded using a JEOL 3011 high-resolution electron microscope with a double-tilt holder and 300 kV of accelerating voltage. Electron-dispersive X-ray spectroscopy (EDS) was also conducted using the same TEM setup. The selected-area electron diffraction pattern [Fig. 1 ▸(*d*)] and the EDS spectrum (see the supporting information) are consistent with the composition [Al_9_(Co,Ni)_4_] and monoclinic crystal structure of the *X* phase, respectively. Furthermore, the columnar crystal morphologies in Fig. 1 ▸(*c*) resemble those found in XRT [Figs. 2 ▸(*k*)–2 ▸(*t*)].

### 2.2. Tomographic data processing   

The XRT data were reconstructed with *TomoPy* (Gürsoy *et al.*, 2014 ▸), a Python-based open-source framework for data processing and reconstruction. First, we normalized the X-ray projection images with white-field images after the dark-field image correction. Normalization helps to compensate differences in the sensitivities and responses of each detector pixel; however, normalization alone is not enough to remove ‘ring’ artefacts, which result from dead pixels in the detector and X-ray beam instabilities. For the removal of ring artefacts, we use a built-in combined Fourier-wavelet filter (Münch *et al.*, 2009 ▸). After normalization and ring artefact removal, the tomographic data were reconstructed via the Gridrec algorithm (Dowd *et al.*, 1999 ▸). Gridrec is based on discrete Fourier transform; further details can be found in Gürsoy *et al.* (2014 ▸). In this way, a stack of 800 2D image slices along the specimen *z* direction (representing a 3D volume) was obtained for each time-step (with a temporal discretization of 20 s).

Further data processing was executed using the Image Processing toolbox in *MATLAB R2016b* (MathWorks, 2016 ▸). We subtracted the image stack of the fully liquefied sample from all other image stacks in order to enhance the contrast between the solid and liquid phases and eliminate any remaining artefacts. The subtracted images were then segmented into solid and liquid phases using a common threshold value for the reconstructed intensity. Morphological operations (*e.g.* image dilation and erosion) were applied to each segmented image to suppress noise arising from segmentation. Finally, the segmented and filtered images were combined to render the 3D microstructures. For the subsequent analysis, the solid–liquid interfaces of the 3D microstructures were meshed or represented by a sequence of triangular faces and vertices. We smoothed the meshed structure via mean curvature flow (Botsch *et al.*, 2010 ▸), so as to remove the staircasing artefacts resulting from the marching-cubes meshing procedure. The following velocity calculations in Section 3.2 rely on accurate face and vertex positions for each time-step. We denote the three vertices of triangle face *i* as 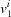, 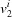 and 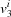. The edges of the triangle face are then defined as 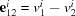, 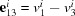 and 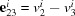. The vertex ordering is consistent for all faces.

### 2.3. Quantitative microstructural analysis   

We extract three pieces of information from our time-resolved XRT experiment: (i) interfacial velocities, (ii) liquid compositions and (iii) two-point autocorrelations of interfacial orientations. Items (i) and (ii) are essential to derive the interface kinetic coefficient 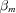 of the habit planes of the *X*-phase crystals, while item (iii) gives us a qualitative measure of the lattice matching between the crystal and its nucleant. Below, we describe the computations required for each parameter in greater detail.

#### 2.3.1. Interfacial velocity calculation   

The local velocity 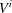 of each face *i* in the mesh is calculated using a nearest-neighbour (NN) algorithm specified by Shahani *et al.* (2016 ▸). The algorithm finds the *NN vertex* in the mesh corresponding to time-step 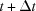 for each *face centroid i* at time-step *t*; then, the velocity of each face at time-step *t* is computed by dividing the distance between the face centroid at time-step *t* and NN vertex at time-step 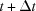 by the time interval 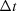. Note that a given crystallographic facet has millions of such triangle faces. The collective *facet* velocity 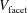 can be computed from the *local* triangle face velocities 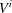 for those faces *i* on the facet using
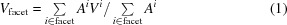
where 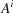 indicates the area of mesh triangle *i*. That is, equation (1) represents a weighted average over all triangles on the facet, where the ‘weights’ are the triangle areas 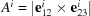 and 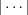 is the vector magnitude. To identify those triangles belonging to a facet, one can cluster triangles based on their interfacial orientation, as was done by Senabulya *et al.* (2018 ▸); alternatively, one can manually crop out facets in the microstructure before using equation (1), as we have done here [see Fig. 6(*b*)].

#### 2.3.2. Composition mapping   

The time-dependent composition of the liquid was extracted from X-ray projection images by analysing the variation of intensity during solidification. Our approach is based on the premise that this intensity can be mapped to composition one-to-one, for small changes in atomic fraction (Husseini *et al.*, 2008 ▸). As shown by Becker *et al.* (2016 ▸), this strategy is viable for both monochromatic and polychromatic sources, provided that the projected intensity has been calibrated against features of known composition. In general, the intensity *I* of a transmitted X-ray beam is sensitive to a number of different factors beyond composition, such as the sample thickness and beam hardening. Therefore, care must be taken to select regions in the projection images with nearly the same sample thickness, to enable accurate comparison between such regions; furthermore, projections of the fully liquefied sample should be subtracted from subsequent projection images to mitigate the effect of beam hardening, as discussed in Han *et al.* (2017 ▸).

After these processing steps, we calibrate the region-averaged X-ray intensities against (i) the solid d-QC and (ii) the equilibrium liquid phase, obtained when the d-QC stops growing at 1247.8 K. In both cases, intensities can be found from the projection images (Han *et al.*, 2017 ▸) and the corresponding compositions from phase diagrams of the Al–Co–Ni system (Yokoyama *et al.*, 1997 ▸). That is, features (i) and (ii) are used as reference points to convert the observed, region-averaged liquid-phase intensities *I* into the total atomic fraction of the heavy elements (Co and Ni) in the liquid, which we denote as 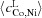. Notably, it is impossible to decouple the individual contributions of Ni and Co within 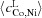; to do so would require a third such calibration point (Han *et al.*, 2017 ▸). Yet it is not so important to make the distinction between Co and Ni since they occupy substitutional sites in the *X*-phase Al_9_(Co,Ni)_4_ lattice (Section 1.1). In practice, the X-ray intensity of the liquid phase should be measured through a designated region of interest (ROI) within which the incident X-ray beam can penetrate without ever encountering the growing crystals. During growth of the *X* phase, only approximately a hundred projections out of a thousand projections (per 180° rotation) can thus be composition mapped. This is because the X-ray path through the liquid phase is blocked by the *X*-phase crystals as the sample rotates in XRT. Nevertheless, these data give us a wealth of information on the time evolution of 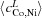, and hence the supersaturation driving force of the parent liquid phase (Section 3.2).

#### 2.3.3. Two-point correlation analysis   

In general, the two-point correlation function is a useful tool to measure and visualize correlations of local attributes [*e.g.* interfacial curvatures and orientations, see Sun *et al.* (2017 ▸)] within the 3D microstructure. The theoretical basis of correlations in the context of materials informatics is well developed (Kalidindi *et al.*, 2011 ▸; Gupta *et al.*, 2015 ▸; Kalidindi, 2015 ▸); here, we want to explore the application of this theoretical machinery to the study of the growing *X*-phase crystals in our work. For instance, to what degree are the interfacial orientations of one crystal correlated to those of the neighbouring crystals? What are the corresponding correlation lengths? Answers to these questions will give us a clue about the role of heterogeneous nucleation in defining crystal orientations. To be more specific, we quantify autocorrelations of facet orientations *u* and 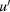 linked by a prescribed vector 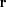 using the Pearson product-moment correlation coefficient 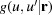, which is valued between 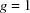 and 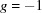 (Hinkley & Cox, 1979 ▸). From computer vision, the local (*i.e.* pixel-wise) orientation *u* in a 2D image can be found as the direction of the image gradient (Russ, 2016 ▸). For 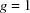, the two given spatial points along the solid–liquid interface are ideally correlated to each other (*i.e*. they possess the same facet orientation, in our case). On the other hand, the two points are anti-correlated when 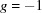. When 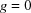, the two points are uncorrelated to each other. Once 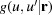 is found for valid vector displacements 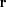, the data are collected on a 2D (or 3D) map with the displacement between a pixel and the centre of the map indicating 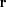, and the value associated with the pixel being the value of *g* at 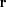.

In practice, we conduct the two-point Pearson autocorrelation analysis in *MATLAB* (MathWorks, 2016 ▸) using the spatial correlation toolbox, release 3.1 (Cecen & Kalidindi, 2015 ▸). We use as input the segmented 2D images taken along the specimen *x*–*z* plane, which is approximately perpendicular to the *b* axis (〈010〉) of the growing *X*-phase crystals [Figs. S2 and S3(*a*) in the supporting information]. The following steps were taken to ensure that meaningful results were obtained. Firstly, the images were cropped so as to exclude the oxide skin and sample holder from our analysis. Next, the images were masked to include only the solid–liquid interfaces [that is, a small thickness was assigned to the interfaces to make them 3D, see Fig. S3(*b*)]. The orientation at each pixel value along the interface was encoded as a continuous range of grey levels [Fig. S3(*c*)]. Finally, the images were padded with zeros on two sides, in order to avoid errors caused by the periodic boundary assumption inherent to discrete Fourier transforms (DFTs, used in the computation of *g*) (Cecen *et al.*, 2016 ▸). The size of the outside padding controls which of the computed two-point statistics are accurate, *i.e.* the correlations are only accurate for vector lengths 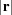 smaller than the padding size. The processed images were passed through the toolbox to output maps of the Pearson autocorrelation function 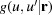 over vector lengths 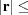 325 µm in the specimen *x*–*z* plane. The computation was repeated for all time-steps over the course of crystallization.

## 3. Results and discussion   

### 3.1. Nucleation dynamics: d-QC versus *X* phase   

#### 3.1.1. Various statistics of nucleation   

**Figure 3:**
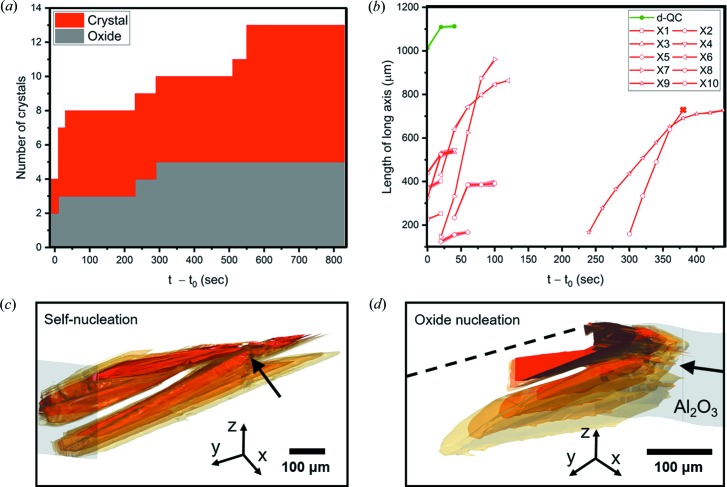
(*a*) Number of nucleated *X*-phase crystals as a function of time *t* following the first nucleation event at time 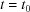. Only those nucleation and growth events that occurred within the tomographic FOV are recorded. Nucleation is heterogeneous and takes place on either existing crystal surfaces or the protective Al_2_O_3_ oxide skin of the sample, with nearly equal probability. (*b*) Length of the ‘long axis’ (parallel to the crystallographic *b* direction) of *X*-phase crystals versus time (red curves). Shown for comparison is the growth trajectory of the d-QC along its long axis 〈00001〉 (green curve). All lengths were measured when the crystals were fully contained within the tomographic FOV except crystal #10; the cross mark at 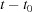 = 380 s for crystal #10 indicates that it grew out of the tomographic FOV during the *in situ* experiment. Measurement errors for crystal (*a*) numbers and (*b*) lengths are minimal and arise from counting statistics. Superimposed 3D reconstructions of *X*-phase crystals that nucleated heterogeneously from (*c*) the existing crystal surface and (*d*) the protective Al_2_O_3_ oxide skin of the sample. Both (*c*) and (*d*) contain four different time-steps with a temporal discretization of 20 s, rendered with decreasing opacity (from opaque red to translucent yellow). The thick arrows in (*c*), (*d*) indicate where the nucleation first occurred and the dashed line in (*d*) indicates where the reconstructed data were cropped for ease of visualization. The grey region represents the Al_2_O_3_ oxide skin.

We show in Fig. 2 ▸ 3D reconstructions of one single d-QC (coloured in green) and multiple *X*-phase crystals (coloured in red). The surrounding liquid phase is rendered in white. The d-QC nucleates at 1259.8 K [Fig. 2 ▸(*a*)] and grows from one side of the protective Al_2_O_3_ skin to the other, along the periodic 〈00001〉 direction (Han *et al.*, 2017 ▸). Only one QC nucleation was detected within the tomographic FOV for the duration of the *in situ* experiment. Nucleation of the *X*-phase crystals takes place later, at 1227.2 K [Fig. 2 ▸(*k*)]. A total of 13 crystals of the *X* phase were captured in the FOV during the tomographic scan, see Fig. 3 ▸. By tracking their formations in 4D, we identify two distinct nucleation mechanisms: (i) *self-nucleation*, wherein intermetallics themselves act as potent nucleation sites for new *X*-phase intermetallic crystals, at large distances away from the specimen surface; (ii) *surface oxide nucleation*, wherein the specimen surface (Al_2_O_3_) acts as a nucleant for the *X* phase. A similar behaviour was reported by Narayanan *et al.* (1994 ▸), Miller *et al.* (2006 ▸) and Terzi *et al.* (2010 ▸) who considered the nucleation of another Al-based intermetallic, β-Al_5_FeSi. We did not detect homogeneous nucleation. The 4D data were thoroughly analysed to classify every *X*-phase crystal according to these two categories, see Fig. 3 ▸(*a*). It can be seen that the total number of nucleation events increases continuously during continuous cooling. This might be because of the strongly anisotropic growth mechanism of the *X* phase, which grows along sharp crystallographic directions (Section 1). Thus, the *X* phase cannot grow into the supersaturated liquid regions that are not in the path of its ‘long axis’. Constitutional undercooling (analysed below) builds up in these liquid regions until it exceeds the necessary nucleation undercooling. At this point, nucleation events are triggered around the existing crystals based on the above-mentioned two mechanisms (i) and (ii). Similar arguments were made by Salleh *et al.* (2017 ▸) to justify their *in situ* observations of repeated nucleation events of faceted Cu_6_Sn_5_ crystals. Fig. 3 ▸(*a*) indicates that, at early times, such heterogeneous nucleation events occur on the surface oxide and the existing crystals with near-equal probability; at long times, there is more surface area on the exposed *X*-phase facets, resulting in a slight bias towards self-nucleation. We expect that these 13 nucleation and growth events are representative of nucleation and growth throughout the entire sample; this is because the alloy melt had been homogenized for around 400 s [Fig. 2 ▸(*j*)] before the first *X*-phase crystals were observed. 3D examples of two different growth mechanisms are illustrated in Figs. 3 ▸(*c*) and 3 ▸(*d*).

Fig. 3 ▸(*b*) shows the length of the ‘long axis’ for each of the nucleated *X*-phase crystals as a function of time. To interpret this plot, we must consider the interactions between the nucleated crystals. Their growth may be physically blocked by each other or the oxide skin (so-called ‘hard collisions’); further elongation along the *b* axis may also be suspended due to a depletion of the available solute in the melt (so-called ‘soft collisions’) (Enomoto *et al.*, 1986 ▸, 1987 ▸; Enomoto, 1991 ▸). The latter occurs when the crystal separation is smaller than the solute diffusion length (see also Section 3.2.1). Due to a combination of both hard and soft collisions, the length of the *X*-phase rods tends to be shorter (on average) than that of the single d-QC, which grows quickly and without any interruption. In contrast, only four *X*-phase crystals are able to extend from one side of the oxide skin to the other. Altogether, by combining Figs. 3 ▸(*a*) and 3 ▸(*b*), it is clear that the *X*-phase rods are *shorter and more numerous* than the d-QC.

**Figure 4:**
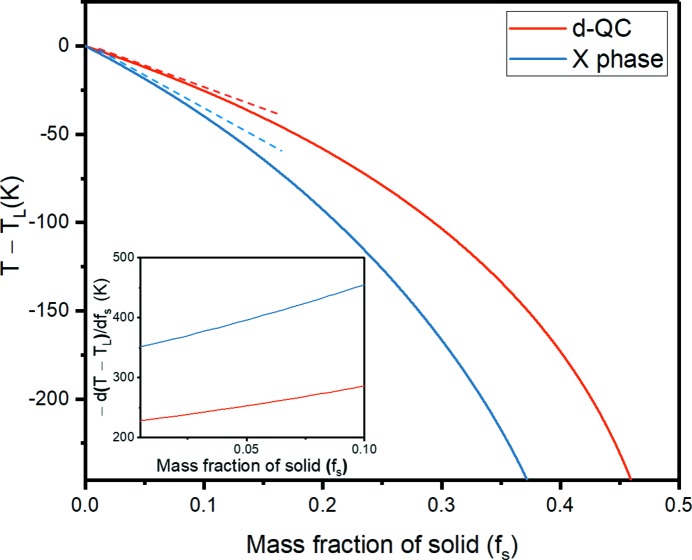
Mass fractions 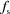 of the solid d-QC (red) and *X* phase (blue) versus relative temperature 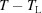, where 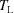 represents the liquidus temperature of either phase. Both curves were calculated using the recent CALPHAD-based assessment of the Al–Co–Ni system from Wang & Cacciamani (2018 ▸). The first derivative 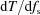 of these two curves in the limit of 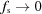 represents the growth restriction factors (GRF) of the d-QC and *X* phase (see inset). The *X* phase has a higher GRF by a factor of around 1.5.

At first glance, the comparatively low nucleation rate (number of nuclei per unit volume per unit time) of the d-QC may seem incongruous with its low solid–liquid interfacial energy γ^SL^ (Holland-Moritz *et al.*, 1993 ▸) and higher nucleation temperature T*. According to classical nucleation theory (Hoyt, 2011 ▸; Kelton & Greer, 2012 ▸), both of these factors tend to increase the nucleation rate over that of the approximant phases. Yet this rudimentary analysis does not consider the influence of solute, otherwise known as *constitutional undercooling*. The development of constitutional undercooling – at the interface of the first crystals to nucleate – starts a ‘wave’ of nucleation events throughout the bulk liquid (Easton & StJohn, 2001 ▸). Some understanding of these constitutional effects can be gained by considering the predicted solidification paths of both d-QC and *X* phases, see Fig. 4 ▸. Both curves were calculated with the aid of *Thermo-calc* software (Andersson *et al.*, 2002 ▸), using a recent thermodynamic assessment of the Al–Co–Ni system as input (Wang & Cacciamani, 2018 ▸). The growth restriction factor (GRF) is defined as the *initial rate of development of the constitutional undercooling* at the solid–liquid interface (Easton & StJohn, 2001 ▸), and can be found directly from Fig. 4 ▸ as 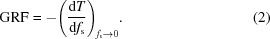
In calculating the GRF, we assume two-phase coexistence only (*e.g.* liquid and *X* phase). Furthermore, we have considered the exact same master alloy composition (Al–8at.% Co–8at.% Ni) as that of our XRT experiment. The inset derivatives in Fig. 4 ▸ indicate that the GRF of the *X* phase is approximately 1.5 times greater than that of the d-QC in the limit of a vanishingly small solid fraction, 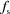. Consequently, a large constitutional undercooling develops in a relatively short growth distance (Salleh *et al.*, 2017 ▸) for the *X* phase, enabling nucleation events to occur closer together (as experimentally observed in Fig. 3 ▸). Thus, due to the growth anisotropy of the faceted *X* phase and its high GRF, it is easier for new crystals to nucleate from the liquid than it is for a single crystal to branch during growth (as for a metal dendrite). In spite of this relatively high nucleation rate, only a few *X*-phase rods can connect to both sides of the oxide skin, due to a high frequency of both hard and soft collisions.

#### 3.1.2. Correlations of interfacial orientations   

**Figure 5:**
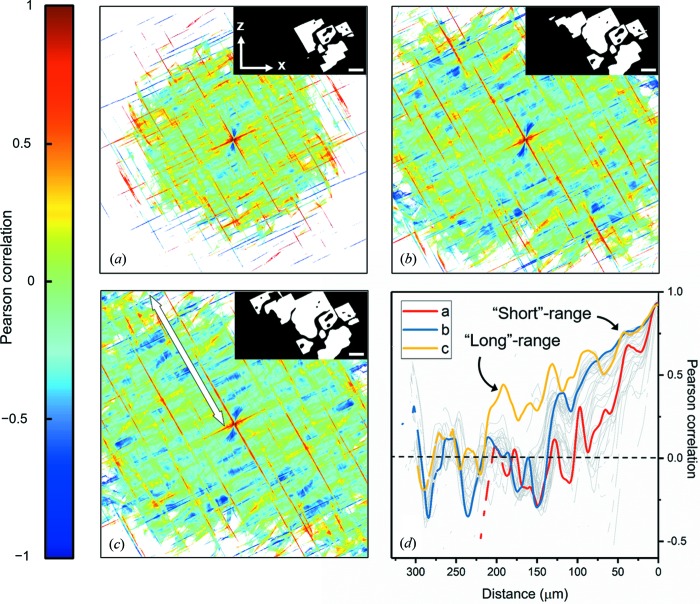
Two-point Pearson autocorrelations of the interfacial orientations within the specimen *x*–*z* plane. The spatial dimension measures 650 µm along each axis. The temperatures and times are as follows: (*a*) 1222.8 K (3020 s), (*b*) 1218.2 K (3300 s) and (*c*) 1213.5 K (3580 s). The Pearson correlation coefficient is undefined (white regions) when the 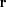-dependent standard deviations [Sun *et al.*, 2017 ▸, equations (28), (29)] of interfacial orientations are zero. More generally, correlations are only valid when the underlying distributions have finite second moments. White–black (*i.e.* solid–liquid) interfaces in the segmented images (inset) were those used to compute the two-point Pearson correlations (see also Fig. S3). The scale bar measures 100 µm in each inset. (*d*) Superimposed two-point Pearson correlations along the white line in (*c*), see text for details. Grey curves correspond to those time-steps (correlation maps not pictured) in between times (*a*), (*b*) and (*c*). The distinction between ‘short’- and ‘long’-range autocorrelations in (*d*) is clarified in Appendix *A* [App appa].

Both the crystal surface and oxide skin play a central role as templates for the nucleating crystals. Lattice matching between crystal and oxide (heteroepitaxy) and crystal and crystal (homoepitaxy) would manifest in a discrete set of allowed orientations for the nucleating crystals. To quantify the alignments in the facet orientations *u*, we calculate correlation maps 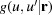 as a function of time within the specimen *x*–*z* plane (see Fig. 5 ▸). Remarkably, this particular plane coincides approximately with the crystallographic {010} plane *for all X-phase crystals*, since they all have ‘long axes’ aligned in the same way; stereographic projections of their long-axis directions are given in Fig. S2. Positive Pearson autocorrelations (*i.e.* 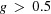) in Fig. 5 ▸ were found along those vector directions 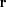 parallel and perpendicular to the facet planes [see also Fig. S3(*c*)]; 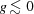 can be seen for all other 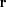. The correlation maps reflect the crystallographic symmetry of the *X* phase in the {010} plane, for reasons discussed below. Positive correlations become more evident and extend over larger distances as the growing *X*-phase crystals approach their fully faceted kinetic Wulff shape (Villain, 1991 ▸; Sekerka, 2005 ▸), that is asymptotically bounded by the slow-growing {100} and {001} facet planes [Fig. 5 ▸(*d*)].

The high Pearson correlations arise from two distinct sources: (i) single facets and (ii) two facets of *neighbouring* crystals (often separated by narrow channels of liquid). The first is responsible for relatively short-range correlations while the latter gives rise to longer-range correlations [Fig. S3(*c*)]. Detailed descriptions of the distinctions between the two are given in Appendix *A* [App appa]. Given the absence of any interfacial curvature, a single facet (i) can possess the same orientation over its length (in 2D), and thus one voxel orientation is correlated to another along the facet itself. The latter case (ii) is related to the epitaxy between crystal and oxide and crystal and crystal (which ‘sets’ the orientations of the neighbouring, nucleating crystals through lattice matching). This may explain why we find correlations of interfacial orientations that extend far beyond the crystal dimension. For example, the maximum facet length [in the inset of Fig. 5 ▸(*c*)] is 180 µm while positive correlations can be seen over much longer distances of 250 µm [Figs. 5 ▸(*c*) and 5 ▸(*d*)]. Yet nucleation does not take place *everywhere* on the surface oxide (Fig. 2 ▸); if it did, the crystals would grow randomly from the oxide skin and radially inward without any such correlations between their interfacial orientations (see, *e.g.*, Salleh *et al.*, 2017 ▸). Rather, nucleation of new crystals takes place *preferentially on or in the vicinity of existing crystals*, in order to relieve the constitutional undercooling as discussed in Section 3.1.1. The rejected solute (Al) from the existing crystals may actually assist the nucleation process by lowering the interfacial energy barrier for heterogeneous nucleation, according to the solute segregation model by Men & Fan (2014 ▸). For instance, the interfacial free energy is smaller for Al (*l*)-Al_2_O_3_ (*s*) compared with that for Ni (*l*)-Al_2_O_3_ (*s*) (Saiz *et al.*, 1999 ▸), and in general decreases as the Al content in the liquid increases (Ni *et al.*, 2014 ▸). This would favour the heterogeneous nucleation of new crystals on Al_2_O_3_ over distances much shorter than the radius of curvature of the oxide skin (∼500 µm). Furthermore, atomic scale observations show that there are up to six layers of partially ordered Al atoms in the liquid at the interface with a structure resembling that of Al_2_O_3_ (Oh *et al.*, 2005 ▸). The result of this solute segregation and epitaxy is that the Al_9_(Co,Ni)_4_ rods point in nearly the same 〈010〉 direction and are perfectly aligned within the {010} plane. Further work is well underway to directly relate the observed growth forms to the chemical structure of the parent liquid phase as well as that of the oxide nucleant, Al_2_O_3_.

### 3.2. Growth dynamics: d-QC versus *X* phase   

Returning to Fig. 2 ▸, it can be seen that the growth of the d-QC along the periodic 〈00001〉 direction is approximately *two orders of magnitude faster* than along the aperiodic 〈10000〉 directions (Han *et al.*, 2017 ▸). Previously, we found that d-QC growth is isotropic – with nearly equal growth velocities along the circumference of the d-QC – whereas its dissolution is markedly anisotropic, the reasons for which are discussed in Han *et al.* (2017 ▸). That is, growth and dissolution do not have time-reversal symmetry. Following the dissolution of the d-QCs (due to segregation effects, see Section 2), we observe the nucleation and growth of the *X* phase. Once nucleated, the columnar morphology of the *X*-phase crystals is similar to that of the d-QC, with long axes aligned along 〈010〉 (see also Fig. 1 ▸). Below we discuss in more quantitative terms the similarities and differences in the growth dynamics of both phases.

#### 3.2.1. Sharp interface model of attachment kinetics   

Once the constitutional undercooling has been relieved, the nucleated crystals must grow to keep up with the cooling rate. To model the growth process, we make use of transition state theory (Wilson, 1900 ▸; Frenkel, 1932 ▸) as follows: at the solid–liquid interface, crystal growth proceeds when the flux of solute from liquid to crystal, 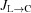, is greater than from crystal to liquid, 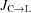. On the other hand, dissolution of crystals takes place when the flux from crystal to liquid is greater than that from liquid to crystal. Stated mathematically, the velocity *V* of a moving solidification front can be expressed as 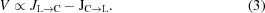
Following thermodynamic convention, 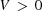 corresponds to growth and 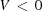 corresponds to dissolution. As the crystal approaches equilibrium with the liquid phase, 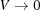. Under weak supersaturation, equation (3) can be expressed as (Jackson, 2004 ▸; Ratke & Voorhees, 2013 ▸) 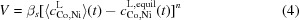
where 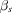 is the kinetic coefficient (as before); the term in square brackets 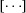 represents the supersaturation driving force (in at.%), 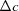; and *n* is an integer exponent that determines the mechanism of cluster attachment [*e.g.* 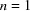 corresponds to ‘normal’ growth on interfacial steps, 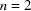 to growth on screw dislocations *etc.* (Ratke & Voorhees, 2013 ▸)]. The supersaturation is defined here as the difference between the *instantaneous*, area-averaged liquid composition, 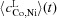, and the *equilibrium* liquidus composition, 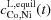, both of which are time *t* (and hence, temperature) dependent during the *in situ* solidification experiment. Equation (4) assumes implicitly that growth is governed by the kinetic contribution to the total driving force. This is a reasonable assumption to make due to the appearance of facets (Figs. 2 ▸ and 6 ▸), which inherently have few positions on the solid surface that are available for attachment (Herlach, 2015 ▸; Libbrecht, 2003 ▸). That is, not every atomic (or cluster) jump from the liquid to the solid will be successful, and thus the growth process will be limited by the kinetics of attachment at the solid–liquid interface. In other words, the kinetic coefficient 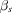 is much less than the ‘diffusive speed’ given by 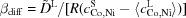, where 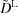 is the interdiffusivity in the liquid phase, *R* is the crystal size, and 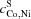 is the composition of Co and Ni in the solid *X* phase (see Appendix *B* [App appb]). A similar assumption was made by Han *et al.* (2017 ▸) in considering the growth of d-QCs in the aperiodic plane, on the basis that all bounding {10000} facets have the same velocity *irrespective of their physical orientation* and are therefore limited by their own intrinsic mobility (as represented by the quantity 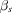).

**Figure 6:**
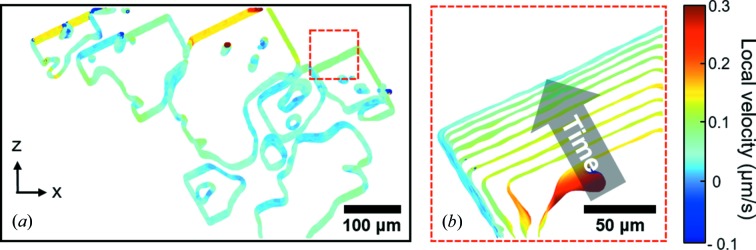
(*a*) Solid–liquid interfaces coloured by the local interfacial velocity at 1216.8 K. Positive interface velocity represents growth and negative velocity represents dissolution. The shown viewpoint is parallel to the specimen *y* axis and the crystallographic 〈010〉 direction. The red dashed box was used to calculate the growth velocity *V* of a single *X*-phase crystal (see text for details). (*b*) Interfacial isochrones with 80 s time increments within the dashed boxed region. The grey arrow indicates the motion of the facet in time. The represented temperatures and times are as follows: 1226.2 K (2820 s), 1224.8 K (2900 s), 1223.5 K (2980 s), 1222.2 K (3060 s), 1220.8 K (3140 s), 1219.5 K (3220 s), 1218.2 K (3300 s), 1216.8 K (3380 s) and 1215.5 K (3460 s).

According to equation (4), we require the (i) growth velocity *V* and (ii) supersaturation 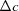 in order to compute the kinetic coefficient 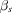 of the *X* phase in the crystallographic {010} plane, and compare with that of the d-QC in the aperiodic {00001} plane (Han *et al.*, 2017 ▸). Both phases have *nearly* isotropic growth rates in the planes perpendicular to the ‘long axis’. In extracting parameters (i) and (ii) from our real-time X-ray imaging data, we must consider carefully the consequence of a relatively high nucleation rate: namely, crystals that grow in close proximity to one another must ‘compete’ for the available solute and thus *growth may stagnate as it becomes solute limited*. That is, the neighbouring crystals act as solute sinks and can dramatically lower the nearby supersaturation. As an example, low facet velocities [indicated by light-blue colours in Fig. 6 ▸(*a*)] are found where the diffusional fields of neighbouring crystals overlap. As the crystals grow, Al is rejected into the melt, accumulating in the open spaces between the crystals. Such solutal interactions have been seen to deactivate the growth of equiaxed grains in metal castings (Badillo & Beckermann, 2006 ▸). For this reason, and to determine the *unbiased* facet velocity, we isolate a freely growing crystal [red dashed box in Figs. 6 ▸(*a*) and 6 ▸(*b*)] that has less of an interaction with other crystals and also enough space to grow further. In addition, we are able to retrieve the instantaneous composition of the bulk liquid 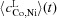 directly from our X-ray projection images, and the equilibrium liquidus composition 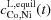 from recent thermodynamic assessments of the Al–Co–Ni system (see Section 2).

**Figure 7:**
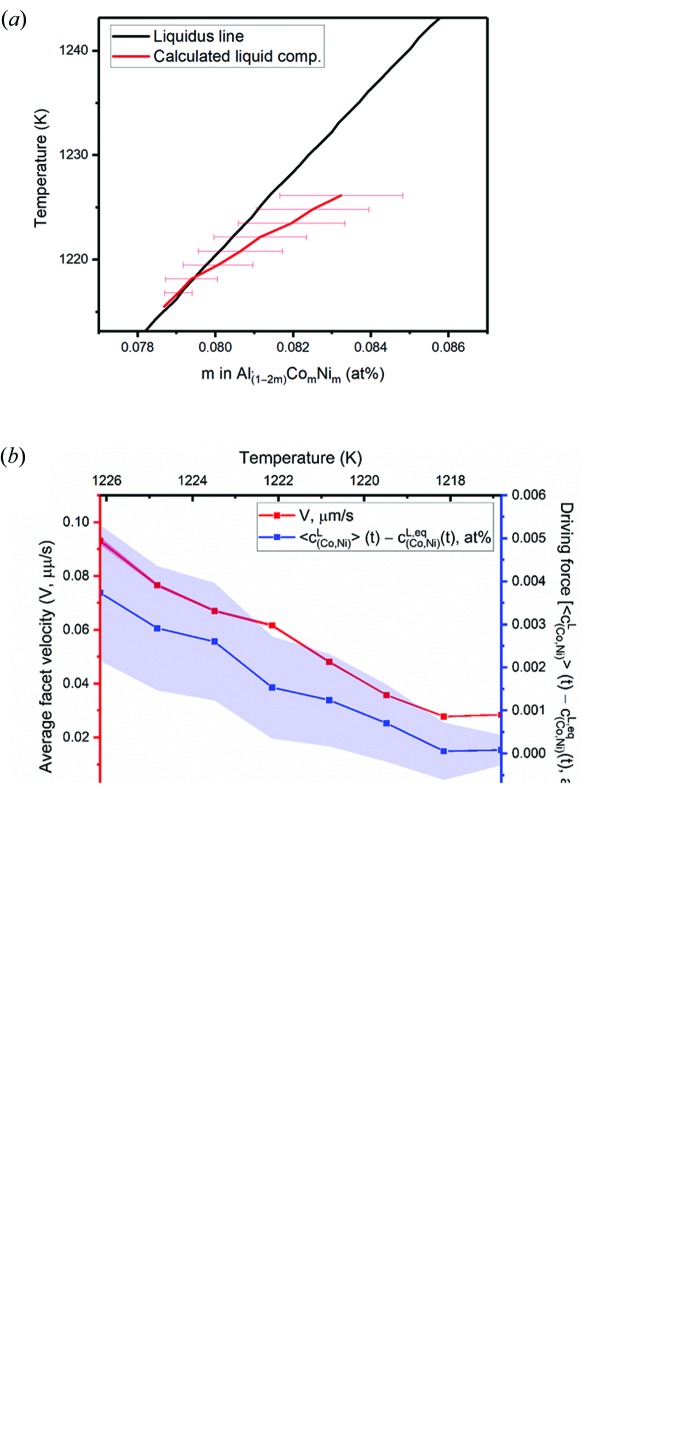
(*a*) Calculated liquid composition (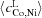, in red) during the XRT experiment, superimposed on a portion of the pseudobinary 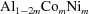 phase diagram (0.074 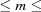 0.088) that shows the equi­librium liquidus curve (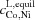, in black). Errors in the calculation of the former are due to slight differences in the sample thickness between independent measurements, which in turn may influence the intensity *I* of the forward attenuated beam (by the Beer–Lambert law, 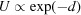, where *d* is sample thickness). The horizontal spacing between the red and black curves represents the supersaturation driving force at a given time and temperature. (*b*) Average facet velocity of a freely growing *X*-phase crystal [in red, see also Fig. 6 ▸(*b*)] and supersaturation (in blue), during the growth process. (*c*) Average facet velocity versus driving force of the d-QC and *X* phase. The slopes give the kinetic coefficient 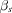 which is associated with the growth process [*i.e.* equation (4) with *n* = 1].

The two compositions are plotted as functions of temperature in Fig. 7 ▸(*a*). At short times (high temperatures) following crystal nucleation, the difference 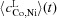 − 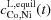 is large, indicating that the liquid phase is highly supersaturated in the elements Co and Ni, whereas at long times (low temperatures) this supersaturation decays to near-zero and hence the two composition curves overlap. Fig. 7 ▸(*b*) indicates that the temporal variations in velocity and supersaturation are comparable with one another during the growth process. By fitting the time-dependent velocity versus supersaturation data to a function of the form given by equation (4), we find the kinetic coefficient 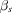 and the temporal exponent *n* to be 1.73 × 10^−3^ cm s^−1^ and 1.0347 for the *X* phase, respectively. An 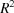 value of 0.974 was obtained, which indicates a good fit of the model to the experimental data. Therefore, the growth process of the *X* phase is dominated by the kinetics of interfacial attachment in the regime of weak supersaturation. More specifically, its growth follows *first-order kinetics* (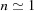) akin to the d-QC (in directions perpendicular to the ‘long’ axis). In addition, we can convert the measured kinetic coefficient in a supersaturated (*s*) matrix, 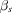, to the more widespread kinetic coefficient in an undercooled (*m*) melt, 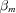, by making use of the liquidus slope. The latter parameter represents the interfacial velocity *V* under unit undercooling (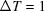
 K). We find 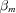 = 4.49 × 10^−7^ cm s^−1^ K^−1^ for the *X* phase in the {010} plane, *which is five times smaller* than that of the d-QC in the aperiodic {00001} plane [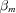 = 2.41 × 10^−6^ cm s^−1^ K^−1^, see also Fig. 7 ▸(*c*)].

One might suppose that the difference in the two kinetic coefficients might be due to different growth temperatures (of approximately 32 K, see also Fig. 2 ▸), since 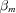 is known to have an Arrhenius-type dependence on temperature *T* (Wilson, 1900 ▸; Frenkel, 1932 ▸; Jackson, 2004 ▸):
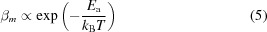
where 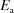 is the activation energy for interdiffusion in the melt and 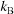 is the Boltzmann constant. For the sake of simplicity, and due to the lack of data on multicomponent melts, we assume that the activation energy for interdiffusion in Al–Co–Ni melt is approximately the same as that of self-diffusion in liquid Al, given by 280 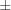 70 meV (Kargl *et al.*, 2012 ▸). By invoking equation (5), we find that the experimentally determined kinetic coefficient 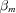 of the *X* phase (4.49 × 10^−7^ cm s^−1^ K^−1^, at its nucleation temperature 1227.2 K) becomes 4.81 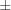 0.08 × 10^−7^ cm s^−1^ K^−1^ at the nucleation temperature of d-QC (1259.8 K). Thus, our crude calculation shows that the ‘temperature-corrected’ 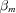 value is almost the same as at a lower temperature. Ultimately, the origin of the different kinetic signatures 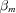 is not a *thermal* one but rather a *configurational* one, as will be explained further in the next section.

#### 3.2.2. Connection between stability and solidification rate   

In general, there are two routes of phase stabilization. One is *kinetic stabilization*, which occurs when crystals are quenched from high temperature to room temperature (see, *e.g.*, Fig. 1 ▸). During the quenching process, the system does not have sufficient time to overcome the energy barrier for phase transformation into other stable phases; thus kinetically stabilized states can be referred to as *metastable states* (Henley *et al.*, 2006 ▸). Another route is thermodynamic stabilization, which consists of two components, energy and entropy. If the former holds true, QCs exist as a ground state of matter, and their stability is determined by the heat of formation 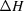 at 0 K. Instead, the stability of QCs can be determined by the entropy term 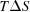 in the expression for the Gibbs energy change, 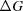. Topological, chemical and phasonic disorders may all contribute to the entropy of the system (Henley *et al.*, 2006 ▸). For instance, QCs which have a broad compositional stability range (*e.g.* the d-Al–Co–Ni phase, investigated in this work) can have large entropic contributions from site-occupancy disorder (either topological or chemical). This might explain why the Ni-rich d-Al–Co–Ni is only stable at high temperature (Lück *et al.*, 1998 ▸).

One way that entropy can be readily incorporated during solidification is if clusters attach to the QC growth front at random. As early as the 1990s, models (see, *e.g.*, Burkov, 1991 ▸) have been proposed for the random packing of decagonal clusters with tenfold symmetry. In this view, the clusters overlap with their neighbours, in the sense that they share atoms with the neighbouring clusters. That is, there are no rules that force clusters into unique arrangements, and hence many possible configurations appear due to the large degrees of freedom on how to join with neighbouring clusters. ‘Errors’ or phason defects are inevitably introduced during the growth process. Such defects increase the phason elastic energy, which in turn is reduced through tile flips, as directly observed *in situ via* HRTEM (Nagao *et al.*, 2015 ▸). In contrast, *those same clusters cannot attach to the periodic approximant crystal at random*, requiring instead extensive cluster rearrangements to maintain the translational symmetry of the underlying lattice. For this process, at least short-range diffusion is necessary. In contrast to bulk diffusion (considered previously), short-range diffusion occurs over only a few interplanar spacings *within* the solid–liquid interface (and thus the growth process is still interface controlled). This explains why some metallic liquids have the ability to deeply undercool (Turnbull, 1950 ▸; Frank, 1952 ▸) and, in our case, why the kinetic coefficient of the periodic approximant *X* phase is about one-fifth of that of the d-QC (see Section 3.2.1). Even if the different growth temperatures *T* between the two phases are accounted for, the kinetic coefficient 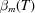 of the periodic approximant is still less than that of the QC. Further support for this idea comes from molecular simulations of QC growth by Keys & Glotzer (2007 ▸). The team showed (in the case of a dodecagonal, one-component) QC that it ‘traps’ icosahedral clusters in the liquid phase with minimal rearrangement; for this reason, the structurally more flexible QC can grow more rapidly than its periodic σ-phase approximant, whose formation would require more extensive local rearrangements (Keys & Glotzer, 2007 ▸). Thus, despite having similar structural motifs (Section 1.1), *the two phases can have very different kinetic signatures*.

That the QC more readily incorporates atomic clusters – pre-existing in the liquid phase – would imply a greater interface width than that of the *X* phase. Indeed, synchrotron-based X-ray diffraction studies on electrostatically levitated metal droplets point to a diffuse interface that effectively ‘blurs’ the distinction between solid QC and liquid (Kelton *et al.*, 2003 ▸). According to the simple model of Tang & Harrowell (2013 ▸), the growth normal velocity *V* is related to this interface width *W* as 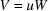, where *u* is the fixed rate at which order increases at any point in the interface. Given the same supersaturation driving force, and holding all else constant, our results would indicate that the interface width of the *X* phase is five times smaller than that of the QC, thus producing a slower growth rate.

Finally, it is worth addressing that phase formation is sensitive to the imposed cooling rate. Here, we cool our master Al–8at.% Co–8at.% Ni alloy relatively slowly, at a rate of 1 K min^−1^ (see Section 2). At a higher undercooling of the liquid phase – wherein (short-range) diffusion is limited – the crystallization of the approximant *X* phase would be kinetically frustrated and thus we would instead expect to see the formation of either QCs and/or metallic glasses. Indeed, fast differential scanning calorimetric (FDSC) studies have recently demonstrated that QCs may serve as an ‘intermediate’ state between liquid and periodic approximant phases (Kurtuldu *et al.*, 2018 ▸). That is, disorder trapping takes place when the growth velocity *V* exceeds the atomic diffusive speed (Boettinger & Aziz, 1989 ▸; Barth *et al.*, 1995 ▸).

## 4. Concluding remarks   

We have performed *in situ* XRT to capture the crystallization of a d-QC and one of its approximants, the so-called ‘*X*’ phase. To the best of our knowledge, our investigation represents the first ever side-by-side comparison of the two solid phases growing from a liquid. On the basis of our dynamical, compositional and correlational analyses, we find that *the d-QC and X phases possess markedly different kinetic signatures despite being crystallographically related.* While growth of the *X* phase is governed by first-order kinetics, in the same manner as for the d-QC, the two solid phases differ with respect to their nucleation and growth rates. A greater constitutional undercooling enables a higher nucleation rate for the *X*-phase crystals. The nucleated, approximant-phase crystals are unable to grow as fast as the d-QC due to ‘soft collisions’ between overlapping diffusion fields. Yet even those *X*-phase crystals that grow freely and away from other *X*-phase crystals have anomalously slow growth rates. This is most likely because extensive cluster rearrangements are necessary to maintain the translational symmetry of the periodic lattice. Meanwhile, the d-QC does not experience as great a kinetic undercooling at the solid–liquid interface since it is able to incorporate the atomic clusters at random. It is for this reason that the measured kinetic coefficient of the *X* phase is about one-fifth that of the d-QC. We expect that this study will improve our understanding of the kinetic factors that drive crystallization of QCs and their related approximants. Further work is well underway to relate the observed growth forms to the chemical structure of the parent liquid phase as well as that of the oxide nucleant, Al_2_O_3_.

## Supplementary Material

Figs. S1, S2 and S3. DOI: 10.1107/S2053273318017114/gv5001sup1.pdf

## Figures and Tables

**Figure 8:**
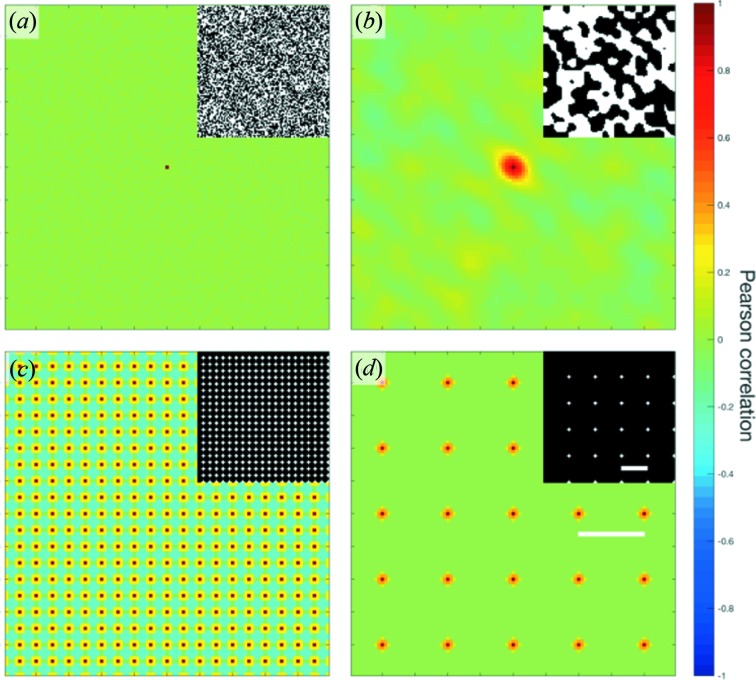
Two-point Pearson autocorrelation maps of four segmented images (inset): (*a*) random noise at the one-pixel scale, (*b*) random noise at the ten-pixel scale, (*c*) square pattern with periodicity of five pixels, and (*d*) square pattern with periodicity of 20 pixels. Features (white) in (*c*)–(*d*) have the same size as 3 pixels. It can be seen in (*a*), (*b*) that the initial descent in the Pearson correlation (from *g* = 1 at 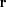 = 0 to 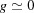 for 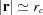) corresponds to the feature size 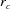; meanwhile, the high-frequency oscillations in (*c*), (*d*) correspond to the distances between features in the images (separated by 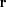). Both scale bars in (*d*) measure 20 pixels.

**Figure 9:**
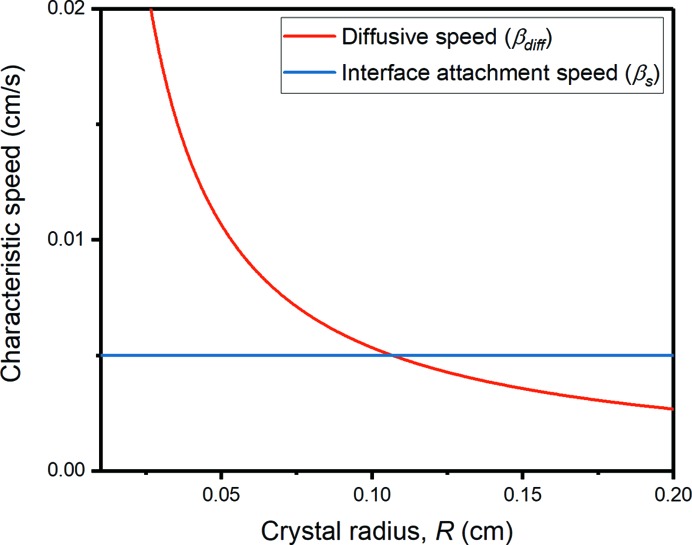
Relationship between crystal radius (*R*) and characteristic speed [interface attachment speed 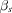 and diffusive speed 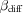, equation (12)]. The interface attachment speed is less than the diffusive speed when the crystal radius is approximately smaller than 0.1 cm (which coincides with the physical sample size). Since crystal sizes observed in our XRT experiment never reach such large dimensions, the growth of the d-QC and *X* phase is dominated by the kinetics of interface attachment. We assume that the surface roughness does not change as the crystal grows, and thus the interface attachment speed is a constant value independent of the crystal radius.

## Appendix A Analysis of correlation regimes

Correlation maps measure the *size* of features, as well as their *distribution* in the microstructure (the feature of interest in our work being the local interfacial orientation). In order to untangle the two in our analysis, we consider four simulated 2D images and their corresponding correlation maps (see Fig. 8 ▸). As can be seen in Figs. 8 ▸(*a*) and 8 ▸(*b*), the length  of the steepest drop in slope in the map of Pearson autocorrelations measures the size of the feature (here, clusters of white pixels). Meanwhile, the period in the high-frequency oscillations in the correlation maps shown in Figs. 8 ▸(*c*) and 8 ▸(*d*) measures the distance between the white clusters (of same size here). Those same long-range correlations are absent in Figs. 8 ▸(*a*) and 8 ▸(*b*) since the two images were randomly generated. In our analysis, we use ‘long range’ to refer to the oscillations outside of the steepest drop; ‘short range’ is just the opposite (Section 3.1.2).

## Appendix B Analysis of crystal growth regimes

In general, the growth of crystals from a supersaturated solution can be influenced by (i) *diffusive transport* through the solution and (ii) *attachment kinetics* at the crystal surface. In order to illustrate the relative importance of both processes, we will consider the growth of a 3D fictitious crystal with an infinite number of facets on all sides (thus rendering it spherical). Diffusive transport can be described by Fick’s second law (Shewmon, 1989 ▸). Assuming that growth proceeds slowly and near equilibrium, the diffusion equation reduces to Laplace’s equation (in spherical coordinates),

where σ denotes the supersaturation field at radial distances *r*,

Equation (6) has the well-known solution (Jackson, 2004 ▸; Ratke & Voorhees, 2013 ▸)

where the constants *A* and *B* are determined by the boundary conditions. Far away from the growing crystal (*i.e.* as ), the supersaturation is given by a baseline value  so that . To solve for the remaining term *A* we require a mass balance condition at the crystal surface , given as

where  is the interdiffusivity in the liquid (L) phase and  is the composition of the solid (S) phase. We also know from equation (4) that the velocity *V* is directly proportional to the driving force . Combining equations (4) and (9) by eliminating *V* gives the mixed boundary condition at the crystal surface,

Substituting equation (8) into equation (10) gives the solution of the diffusion equation and the velocity *V* as

where the term  is the ‘diffusive speed’, which we define as

In the limit as , the growth velocity in equation (11) becomes  (which is independent of the kinetic coefficient ) and thus describes purely diffusion-limited growth. In the opposite limit, we have  which is valid for interface-limited growth; note this expression is analogous to equation (4) in the main text. Thus, the dominant mechanism of crystallization depends on the relative magnitudes of the characteristic speeds  and . We can compare the two using some realistic parameters. As in Section 3.2.2, we assume that  is equivalent to the self-diffusivity of Al in its melt ( cm^2^ s^−1^ at 1020 K) (Kargl *et al.*, 2012 ▸); we let *R* be  cm; and we take  to be approximately 0.15, based on our compositional analysis. The calculated  is  cm s^−1^ which is far greater than typical values seen for  (*e.g.*  cm s^−1^ for the d-QC). Thus,  is always greater than  in the range of our crystal size. It follows that diffusion is fast (for the crystal sizes so-investigated) and hence interfacial attachment is the rate-determining step in crystallization. Of course, as crystallization proceeds and the crystal size *R* increases, the diffusive speed [equation (12)] lags and the process will eventually become diffusion limited (see Fig. 9 ▸).

While the preceding analysis illustrates concisely the two regimes of crystal growth, it is important to bear in mind that it makes a number of simplifying assumptions: (i) the supersaturation is certainly not a constant value but instead changes with time until it is depleted and growth is finished [*cf.* Fig. 6 ▸(*a*)]. (ii) Only growth of a single crystal is considered here. Instead, the multi-particle diffusion problem (Ratke & Voorhees, 2013 ▸) is likely more realistic since it accounts for the ‘soft collisions’ that are seen at high nucleation rates [*i.e.* nearby crystals act as solutal sinks, *cf.* Fig. 5 ▸(*a*)]. (iii) We do not account for the influence of capillarity, in which the interfacial composition  depends on the interfacial curvature [or weighted mean curvature for a faceted surface (see Taylor, 1992 ▸; Roosen & Carter, 1998 ▸)]. This assumption may not be so detrimental given the effects of attachment kinetics are usually much greater than the effects of the Gibbs–Thomson mechanism (see, *e.g.*, Libbrecht *et al.*, 2002 ▸). (iv) Another potential shortcoming is that our analysis does not account for the deposition of latent heat in the transition from liquid to solid, which may change the supersaturation locally (especially if thermal diffusion is slow). (v) Lastly, for the sake of simplicity, we neglect the strong crystallographic anisotropy of the rod-like *X*-phase crystals. We would need to invoke the conformal mapping of the Shwarz–Christoffel type (Trefethen, 1980 ▸) in order to solve equation (6) under realistic boundary conditions and ultimately for the orientation-dependent facet velocities. Indeed, including all effects from (i) to (v) would require substantial numerical modelling, which is beyond the scope of the present investigation.
