# Supplementary material for: A side-by-side comparison of the solidification dynamics of quasicrystalline and approximant phases in the Al–Co–Ni system
Source: Acta Crystallogr A Found Adv. 2019 Feb 6;75(Pt 2):281–96. doi: 10.1107/S2053273318017114 (PMC6396399; doi:10.1107/S2053273318017114)
Supplement: Supplementary file 1 [file a-75-00281-sup1.pdf]

FOUNDATIONS
ADVANCES

Volume 75 (2019)

Supporting information for article:

A side-by-side comparison of the solidification dynamics of quasicrystalline and approximant phases in the Al-Co-Ni system

Insung Han, Xianghui Xiao, Haiping Sun and Ashwin J. Shahani

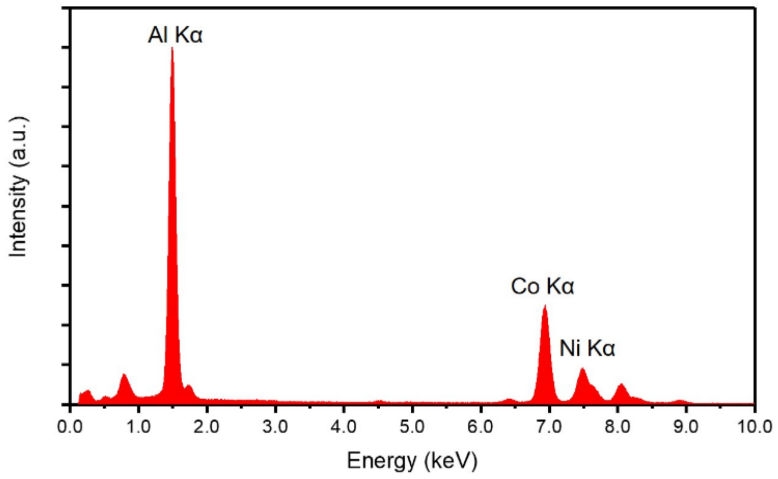

Figure S1 Energy dispersive spectroscopy (EDS) data collected on the as-synthesized X phase. The average atomic percent of Al, Co and Ni ratio from multiple TEM EDS measurements was 67.4 $\pm$ 1.7 at%, 24.0 $\pm$ 1.1 at% and 8.7 $\pm$ 0.8 at%, respectively. The average EDS values correspond well with the known X phase composition, $Al_9(Co,Ni)_4$. The small peak at 8.05 keV corresponds to the Cu TEM grid.

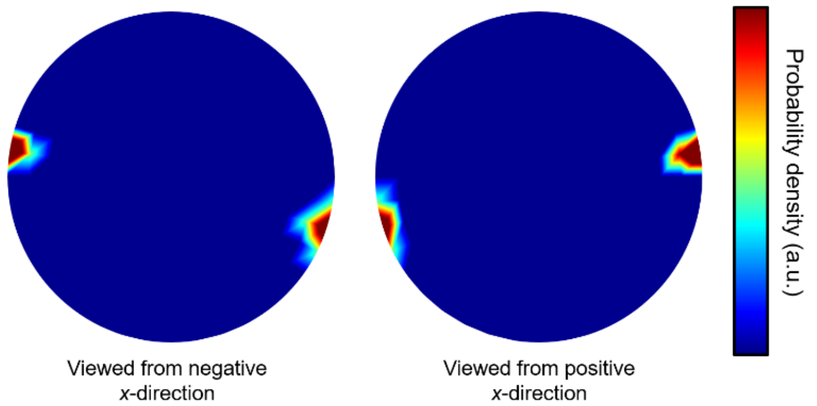

Figure S2 Stereographic projections of the long axes of the X phase crystals. Shown are two opposing viewpoints in the specimen frame, the negative x-direction (left) and the positive x-direction (right). The sharp peaks in both probability distributions indicate the strog degree of alignment of the crystals.

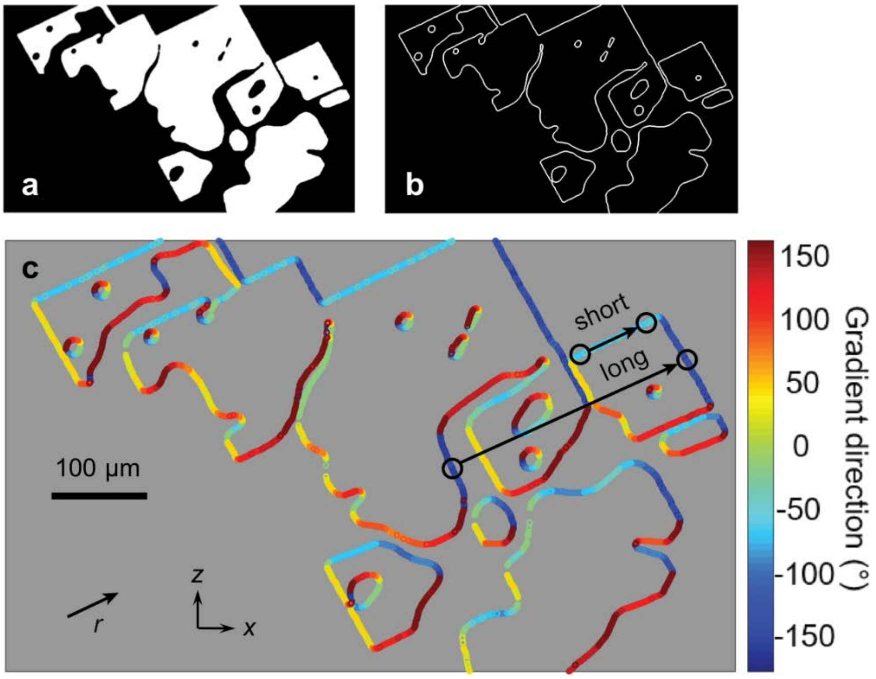

Figure S3 Processing steps for correlation analysis. (a) Segmented image taken along the specimen x-z plane, which is nearly perpendicular to the crystallographic {010} plane of all growing X phase crystals (see also **Fig. S2**). (b) Mask image that only includes the solid-liquid interfaces of (a). (c) The orientation of each pixel belonging to the interface in (b), encoded as the direction of the image gradient (measured counter-clockwise from the positive x-axis, see colorbar). Note that opposite sides of a given crystal have orientations that differ by 180°. The arrows indicate both short-range (denoted "short") and long-range ("long") autocorrelations $g(u, u'|\vec{r})$ of facet orientations u along the prescribed vector $\vec{r}$. The temperature (time) corresponds to 1213.5 K (3580 s).
